# Clinician-Led Development and Feasibility of a Neural Network for Assessing 3D Dental Cavity Preparations Assisted by Conversational AI

**DOI:** 10.3390/dj13110531

**Published:** 2025-11-13

**Authors:** Mohammed El-Hakim, Haitham Khaled, Amr Fawzy, Robert Anthonappa

**Affiliations:** 1 Dental School, University of Western Australia, Perth 6009, Australia; amr.fawzy@uwa.edu.au (A.F.); robert.anthonappa@uwa.edu.au (R.A.); 2 School of Engineering, Edith Cowan University, Perth 6027, Australia; h.khaled@ecu.edu.au

**Keywords:** dental education, artificial intelligence, neural networks, 3D dental cavity, ChatGPT, clinician led AI

## Abstract

**Introduction:** Artificial intelligence is emerging in dental education, but its use in preclinical assessment remains limited. Large language models like ChatGPT^®^ V4.5 enable non-programmers to build AI models through real-time guidance, addressing the coding barrier. **Aim:** This study aims to empower clinician-led, low-cost, AI-driven assessment models in preclinical restorative dentistry and to evaluate the technical feasibility of using a neural network to score 3D cavity preparations. **Methods:** Twenty mandibular molars (tooth 46), each with two carious lesions, were prepared and scored by two expert examiners using a 20-point rubric. The teeth were scanned with a Medit i700^®^ and exported as .OBJ files. Using Open3D, the models were processed into point clouds. With ChatGPT’s guidance, the clinician built a PointNet-based neural model in PyTorch, training it on 20 cases and testing it on 10 unseen preparations. **Results:** In training, the model achieved an MAE of 0.82, RMSE of 1.02, and Pearson’s r = 0.88, with 66.7% and 93.3% of the predictions within ±5% and ±10% of the examiner scores, respectively. On the test set, it achieved an MAE of 0.97, RMSE of 1.16, and r = 0.92, with 50% and 100% of scores within ±5% and ±10%, respectively. These results show a strong alignment with examiner scores and an early generalizability for scoring preclinical cavity preparations. **Conclusions:** This study confirms the feasibility of clinician-led, low-cost AI development for 3D cavity assessment using ChatGPT, even without prior coding expertise.

## 1. Introduction

Artificial intelligence (AI) is rapidly transforming healthcare education by enabling new approaches to personalized learning, objective assessment, and data-driven decision-making [1,2]. In dental education, AI applications have expanded to include diagnostic image interpretation, the automated evaluation of clinical work, intelligent tutoring systems, and even adaptive curriculum customization to individual learner needs [3,4,5]. For example, AI can assist in interpreting radiographs, grading procedures, and tailoring learning feedback to students [5].

However, a significant implementation gap persists. Most dental educators lack the technical expertise necessary to develop or customize AI systems, since skills like computer coding and machine learning workflow design lie outside typical clinical or academic training in dentistry [3,6]. As a result, AI tools are often outsourced to non-clinicians, risking a misalignment with educational goals [3,4].

Creating AI models capable of processing three-dimensional (3D) dental data remains especially challenging [7]. AI tools often lack transparency, making them hard to interpret and trust, underscoring the need for explainable AI and governance [8].

The emergence of large language models (LLMs), such as ChatGPT, offers a novel way to begin bridging this gap. These AI-driven assistants enable natural language interactions in which non-programmers can collaboratively develop code, receive real-time feedback, and iteratively troubleshoot errors in a conversation format.

Recent studies confirm that LLMs can generate Python code to build, train, and optimize AI models, enabling non-programmers to create functional neural networks. Nelson et al. showed that ChatGPT could produce malware detection code with a high accuracy, while An et al. demonstrated its full machine learning pipeline support for environmental science tasks through a step-by-step interaction [9,10]. A review by Sallam highlights ChatGPT’s role in democratizing access to AI tools, especially for education and research [11]. These findings support LLM’s potential as a coding assistant beyond language tasks, enabling guided model development.

Despite the growing application of AI across various fields, the use of large language models (LLMs) for AI development remains largely unexplored in dentistry. To date, no published studies have documented a dental educator independently constructing a neural network model using conversational AI as a real-time coding tutor. Furthermore, there is no existing evidence of AI being applied to evaluate three-dimensional (3D) preclinical cavity preparations in dentistry. The literature identifies a significant gap in clinician-led AI development, with recommendations emphasizing the advancement of AI tools capable of assessing manual skills within preclinical simulation settings to provide objective, real-time feedback aligned with expert performance standards and educational goals [12]. The present study directly addresses these identified gaps by combining clinical expertise with conversational AI guidance to develop a functional AI model, representing a novel and potentially transformative approach to AI-driven dental education.

## 2. Aims

This feasibility study aims to empower clinician-led, low-cost, AI-driven assessment models in preclinical restorative dentistry and to evaluate the technical feasibility of using a neural network to score 3D cavity preparations.

## 3. Methodology

### 3.1. Cavity Preparation

This study has been exempted from human ethics approval from The University of Western Australia IRB with reference 2025/ET000746, 2025-07-29. We prepared 30 cavity preparations on standardized simulation teeth as part of a preclinical dental assessment study. No specimens were excluded. Before any model training, we prospectively designated 10 preparations as the test set by manual random allocation. Allocation was performed by placing each fully scored specimen, together with its scoring sheet, in an individual sealed envelope, physically shuffling the envelopes, and selecting 10 at random for the test set to be scanned after initial model development. The remaining 20 preparations constituted the training set. All examiner scoring was completed prior to model development, and examiners were blinded to whether a specimen was assigned to the training or test set.

To avoid any mix-up between training and testing, we set aside the 10 test cases in advance and stored training and test sets in separate folders with unique file names. We also checked the files so that there were no duplicates or near-duplicates across the two sets.

All teeth used were mandibular first molars (tooth 46). Each tooth contained two non-joining simulated carious lesions: one occlusal and one mesial. Preparations followed conservative design, preserving structure while removing caries.

During mesial box preparation, the aim was to establish appropriate clearance from adjacent teeth in the buccal, lingual, and gingival directions. The artificial caries extended close to the simulated pulp chamber to replicate the clinical challenge of deep lesion management. While preparing the teeth, some were deliberately made less conservative, some areas of contact with the adjacent teeth were left intentionally, and some caries was left to create scoring variation.

### 3.2. Scoring Training Dataset

Each preparation was assessed independently by two authors who are experienced restorative dentistry examiners for cavity preparation tasks using a structured rubric with a total score of 20 points. The rubric allocated up to 9 points for complete caries removal, 2 points each for buccal, lingual, and gingival clearance, 2 points for the preservation of tooth structure between the cavities, 1 point for the avoidance of cusp weakening, and 2 points for the absence of undermined enamel at the cavity margins. Any preparation that resulted in simulated pulp exposure was given a score of zero according to the rubric. Additionally, iatrogenic damage to adjacent teeth resulted in score deductions, with up to 2 points deducted for minor damage (<1 mm), and up to 4 points for moderate damage (1–2 mm). To ensure the reliability of the expert scoring used for model training and evaluation, inter-examiner agreement was assessed using the Intraclass Correlation Coefficient (ICC 2,1). The ICC between the two examiners across 20 cavity preparations was 0.90, indicating an excellent consistency in scoring. This high level of agreement validates the use of these scores as a ground truth reference. Hence, for all subsequent analyses, the average of the two examiners’ scores was used as the final reference score for each case.

### 3.3. Scanning Cavity Preparations

All prepared teeth were then scanned using the Medit i700 intraoral scanner manufactured by Medit Corp, South Korea. Following scanning, the data were processed using the Medit Link software V3.4.1. The tooth selection tool within the software was used to crop the scan, isolating only the prepared tooth (tooth 46) and its adjacent teeth. This isolated the region of interest and removed unrelated structures. Final 3D models were exported in .OBJ format, preserving surface mesh detail. These files served as the basis for dataset generation for the AI model training.

### 3.4. Neural AI Model Development

To prepare the dataset for AI processing, the large language model ChatGPT was employed to guide the clinician through data transformation steps using Python code. All scripts were written by ChatGPT and implemented by the clinician in a Jupyter Notebook interface. The environment was managed using the Anaconda distribution v 2025.06, which allowed for the installation and configuration of required libraries in a user-friendly framework accessible to non-programmers.

The following are examples of prompts used during the conversation with the LLM AI: “I need step by step guidance so I can do the coding and programming for this AI model. I have limited but acceptable IT experience. Can you guide me through it and teach me?” ChatGPT said, “I’ll guide you step by step to build an AI model using Open3D for automated dental cavity grading. Since you have limited but acceptable IT experience, I’ll break everything down in a structured way.”

Another example prompt was the following:

“Which gpt model is good at 3D image analysis for dental cavity preparation assessment.”

ChatGPT said the following:

“For 3D image analysis, particularly for analyzing OBJ files or other 3D model formats, GPT models alone are not ideal, as they primarily handle text based reasoning and processing. However, multimodal AI models or specialized AI tools are better suited for 3D image analysis. Recommendation for Your Needs: Since you’re working on AI based dental cavity preparation assessment, you should consider: PointNet or Open3D for direct analysis of OBJ files.”

Another example of the conversation is as follows:

“I need a step by step guidance so i can do the coding and programming for this ai model, i have limited but acceptable it experience, can you guide me through it and teach me.”

ChatGPT said the following:

“I’ll guide you step by step to build an AI model using Open3D for automated dental cavity grading. Since you have limited but acceptable IT experience, I’ll break everything down in a structured way.”

ChatGPT even gave step-by-step instructions on installation of the required software in the below example.

“I need to install anaconda please guide me into the steps.”

ChatGPT said the following:

“Excellent! Let’s go through a clear, step by step installation of Anaconda, so you can start using Jupyter Notebooks for your AI project.”

A larger sample of the conversation with ChatGPT is available online for the reader; please see the Data Availability section of the manuscript.

**Figure 1:**
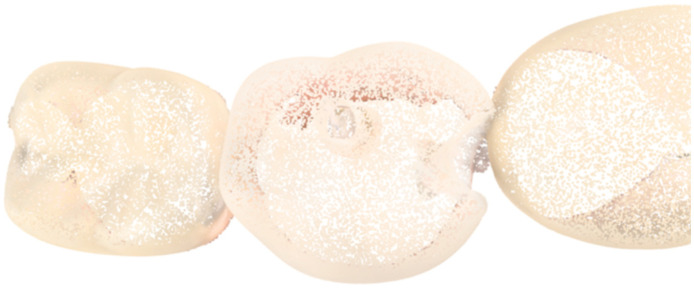
Point cloud file visualization. Example Jupyter Notebook cell showing loading of .OBJ file, conversion to point cloud, and visualization for data inspection.

A key part of the data preparation process involved converting the .OBJ files into point cloud format. This was achieved using the Open3D library V0.19, an open source Python toolkit designed for 3D data processing. Open3D supports tasks such as reading mesh files, generating point clouds, applying transformations, and visualizing 3D structures. In this study, Open3D was used to read each .OBJ file and convert it into a uniformly down-sampled point cloud of 100,000 points. A point cloud represents the surface of a 3D object as a collection of discrete points in three-dimensional space and is the preferred input format for many deep learning models in 3D object recognition. An example of a point cloud visualization is shown in Figure 1.

**Figure 2:**
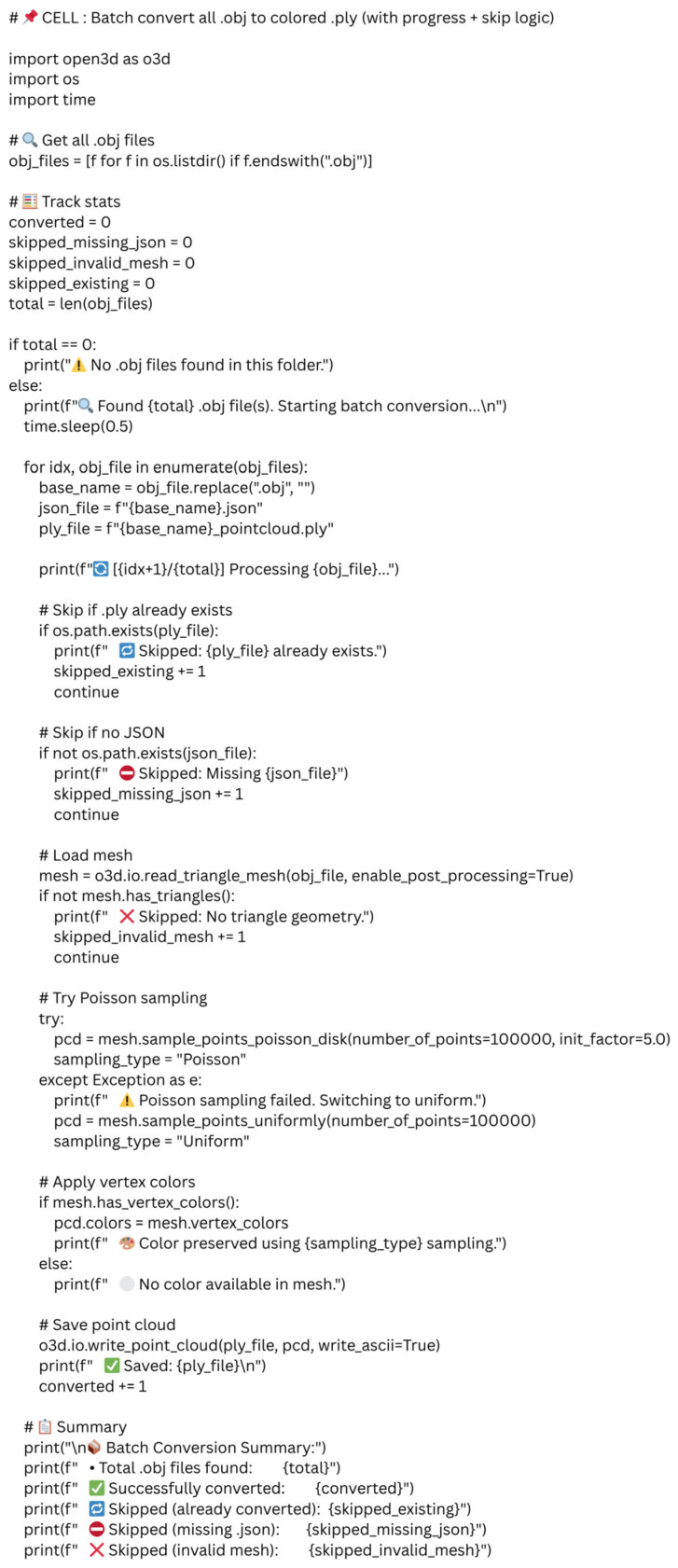
A Jupyter notebook code cell as provided by ChatGPT to convert OBJ files to point cloud.

Supported by the conversational AI’s real-time explanations, the clinician was guided through each step of the data preparation process. ChatGPT provided Python code, which was transferred directly into the Jupyter Notebook environment, allowing the clinician to execute the full workflow without prior coding experience. The guidance included importing the necessary libraries, loading the .OBJ files, converting them into point clouds, visualizing the 3D data, and saving the processed outputs in a standardized format. This structured, step-by-step process enabled the clinician to independently perform complex 3D data handling within a user-friendly programming interface. An example of the code provided by ChatGPT to convert .OBJ files to point clouds is provided in Figure 2.

The application converts it to a point cloud of exactly 100,000 points and then sends that point cloud to the model. Before sampling points, the mesh is moved so it is centered and resized to a common scale so that every case is treated the same. To make results repeatable, we use a fixed seed which is just a single number that makes any random choice happen the same way each time. Using this fixed seed means that the same OBJ file will always produce the same 100,000 points and the same score.

### 3.5. Linking Scores to 3D Data

**Figure 3:**
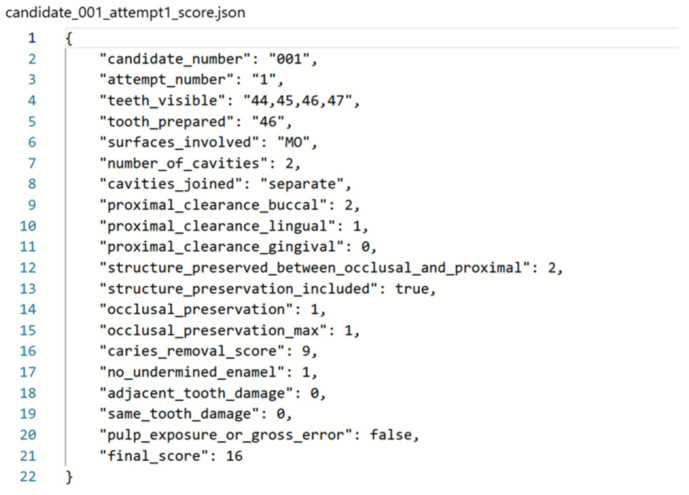
Rubric-linked JSON schema example. Structure of the JSON labeling file used to annotate cavity preparations with nine clinical criteria for model training and scoring.

Each 3D model was linked to a corresponding metadata file in JSON format, which stored the detailed rubric scores assigned to each case. An example of a JSON file with rubric scores is shown in Figure 3. Conversational AI provided Python code to generate these JSON files and populate them with the appropriate values, including per criterion scores and total marks. Scripts ensured each point cloud matched its JSON label file. This pairing enabled the training of a supervised learning model in which the AI would learn to predict rubric-based scores from the 3D geometry of the preparation.

### 3.6. Model Training

In the following step, the conversational AI provided instructions for constructing a custom data loader using PyTorch V2.6, a popular deep learning framework. The PyTorch dataset class was designed to load, normalize, and batch the point cloud data along with their corresponding labels from the JSON files. The LLM provided code and debug support for the data loader to ensure compatibility and stability.

The model used in this study was built using a special type of neural network called PointNet [13], which is specifically designed to understand and process point cloud data made up of thousands of individual surface points representing the shape of the prepared tooth. This model could learn from the 3D structure by passing the data through a series of mathematical steps, ending in what are called fully connected layers.

The learning process occurred over multiple epochs. An epoch refers to one complete cycle, where the model looks at every example in the training dataset once. After each cycle, the model produced a set of predicted scores, which were compared to the actual scores given by human examiners. By comparing its predictions to expert assessments, the model learned to improve its performance step by step over time.

The training of the network was performed to minimize the mean absolute error, measured in rubric points. Training was conducted in two blocks.

We trained the network in two blocks. An initial 50 epochs were run; because training loss (MAE in rubric points) continued to decline, we extended training by a further 50 epochs.

**Figure 4:**
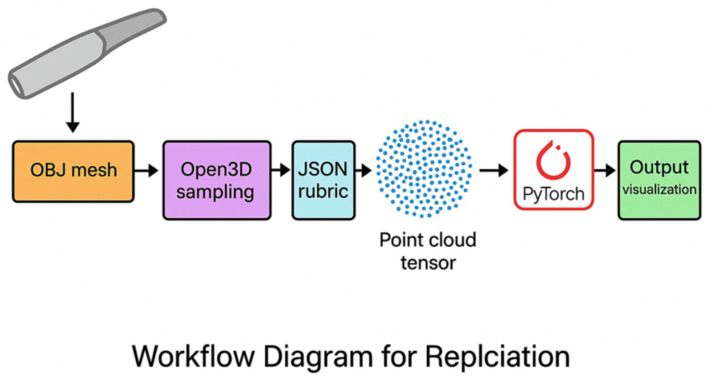
Workflow diagram for replication. Visual outline of the full AI development pipeline from 3D scan to score output: OBJ mesh → Open3D sampling → JSON rubric → point cloud tensor → PyTorch neural net → output visualization.

To ensure that the best version of the model was saved, a process called checkpointing was used. During training, the model’s accuracy was monitored, and whenever the model performed better than before on an epoch run, that version was automatically saved separately. This allowed for keeping the most accurate and reliable version of the model for future applications. It also allows future training scalability. This workflow aligns with best practices in explainable AI and educational modeling [14]. A simplified workflow diagram for creating the model is shown in Figure 4.

### 3.7. Model Evaluation and End User Inference

After 100 epochs of training, the model was tested on a pre-allocated additional set of 10 cavity preparations (the testing dataset), all prepared and marked using the same scoring rubric and methodology by two author examiners; scoring was performed prior to allocation to this group.

At testing, the user uploaded an OBJ mesh, and the application automatically converted it to a point cloud with 100,000 points using the same preprocessing as in training, then generated the score. This automation simulates an end user workflow in which only a standard scanned OBJ is required. The model consumed the resulting point cloud and produced predicted rubric scores, which we compared with the average scores assigned by the two examiners to evaluate accuracy, generalization, and alignment with expert clinical assessment.

The primary outcome was model alignment with examiner total rubric scores (MAE, RMSE, Pearson’s r). Per criterion values are reported descriptively and were not powered or designed for validated sub-score inference in this pilot.

Given the pilot nature of this work, a classical hypothesis testing power analysis for a neural network was not applicable. Instead, we adopted a precision-based feasibility rationale: the goal was to obtain initial estimates of alignment (e.g., MAE, r) with broad 95 percent confidence intervals sufficient to inform effect size and variance assumptions for a planned multi-site study. The present sample (20 training, 10 independent test) is not intended to establish generalizable validity; it is intended to demonstrate feasibility and provide variance estimates to plan a larger trial.

## 4. Results

### 4.1. Training Performance

**Figure 5:**
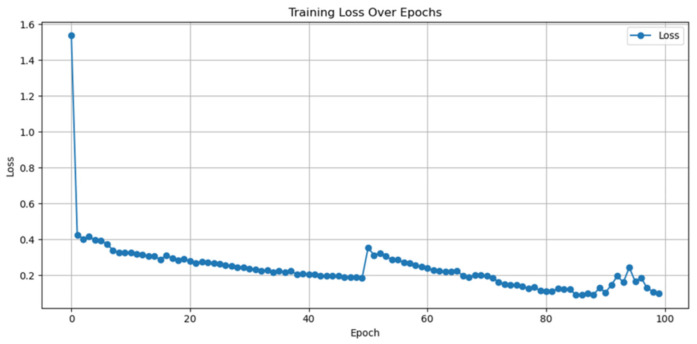
Training loss and training mean absolute error across 100 epochs. The model was trained in two blocks of 50 epochs each. The small rise at epoch 51 marks the start of the second block, after which the metrics continue to improve.

In the training phase, training loss, defined as the mean absolute error (MAE) in rubric points computed on the training set each epoch, decreased steadily over time. It fell from approximately 1.52 at the start to around 0.20 by epoch 50. When training resumed for the second block, there was a brief step to about 0.33 at epoch 51, followed by a further improvement to roughly 0.12 by epoch 100. Epoch 85 was automatically saved as the best model checkpoint and showed a training loss of 0.091. These values describe the training performance only; the evaluation metrics are reported separately. The full training loss curve is shown in Figure 5. We saved a checkpoint at every epoch and restored the weights from the epoch with the lowest training MAE (“best-epoch” checkpointing). We did not carve out a validation split from the 20 training cases in this pilot to preserve the sample size; therefore, the curve we report is for the training loss only.

**Figure 6:**
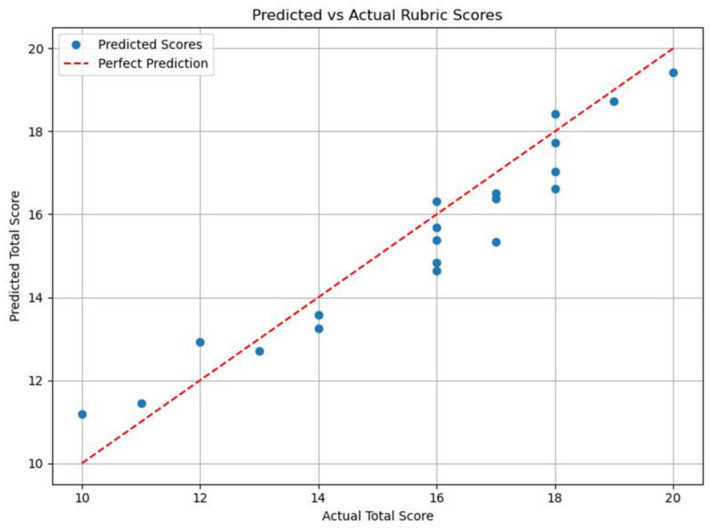
Predicted total score from model vs. actual (human) total score of the training dataset.

The model was evaluated on 20 cavity preparations using the average scores of two expert examiners as the reference. Following the 100 training epochs, with the model automatically saving epoch 85 as the best model based on loss, it demonstrated a strong alignment with human judgment, achieving a mean absolute error (MAE) of 0.82, a root mean squared error (RMSE) of 1.02, and a Pearson correlation coefficient (r) of 0.88, reflecting a high degree of linear association. The model’s predictions deviated, on average, by less than one point from the examiner scores. For the same 20-case set, Spearman’s rank correlation was ρ = 0.967, and Lin’s concordance was ρc = 0.944, with Cb = 0.979, indicating a high absolute agreement relative to the 45° line. Bland–Altman analysis showed a mean bias of −0.39 rubric points, with 95 percent limits of agreement from −1.87 to +1.09 rubric points. The scores predicted from the model are presented below plotted against the examiner average scores (actual scores) in Figure 6.

**Figure 7:**
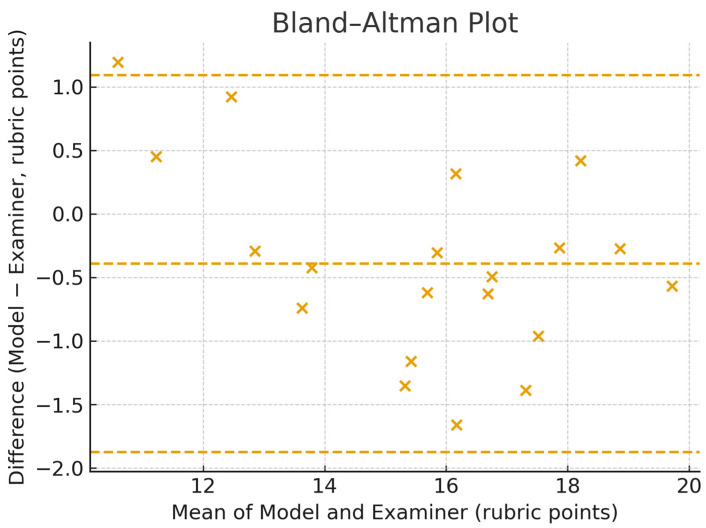
Bland–Altman plot of agreement between model predictions and examiner averages on 20 training cavity preparations.

The Bland–Altman plot of agreement between model predictions and examiner averages for the 20 cavity preparations is displayed in Figure 7.

Accuracy was further assessed based on educationally acceptable thresholds: 66.7% of predictions were within ±1 point (±5% of the 20-point total rubric), and 93.3% were within ±2 points (±10%), demonstrating a strong consistency with expert scoring and supporting the model’s early promise. As neural networks improve with larger datasets, accuracy should improve with further training [15].

Presented in Table 1 is the detailed scoring per rubric item, comparing both the predicted scores (model scores) and the human scores (actual scores) for the training set.

### 4.2. Preliminary Evaluation

To assess generalizability, the model was evaluated on a testing set of 10 cavity preparations that had not been used during training. Each preparation was independently scored by two expert examiners using the same structured rubric. The inter-examiner reliability was calculated using ICC (2,1), yielding a value of 0.89, which reflects a strong agreement. Therefore, the average of both examiners’ scores was used as the reference standard for evaluating model predictions. At test time, the application converted each uploaded OBJ to a 100,000 point cloud using the same automated preprocessing as in training, and the model produced rubric scores from this point cloud.

**Figure 8:**
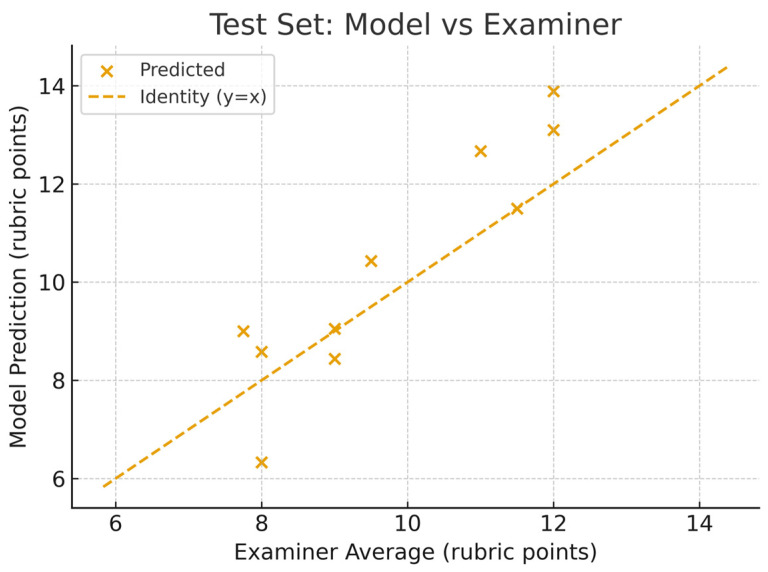
Predicted total score from model vs. actual (human) total score of the testing dataset.

**Figure 9:**
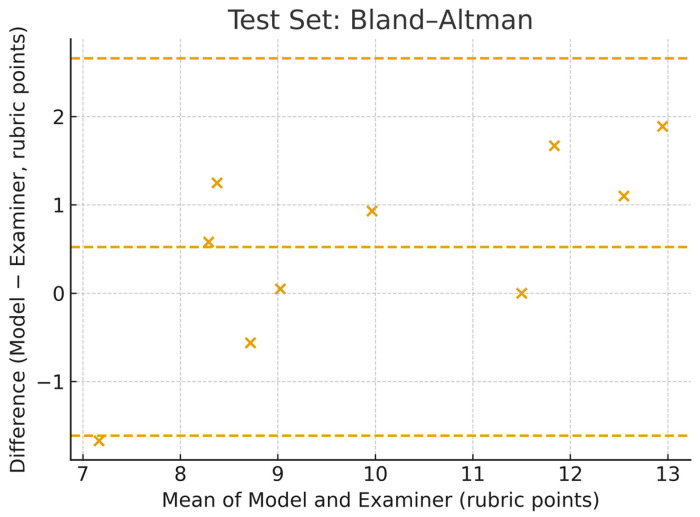
Bland–Altman plot of agreement between model predictions and examiner averages on 10 test cavity preparations.

On the testing set, the model achieved a mean absolute error (MAE) of 0.97, a root mean squared error (RMSE) of 1.16, and a Pearson correlation coefficient (r) of 0.92, indicating a strong linear relationship with the expert examiner scores. In addition to Pearson’s r, we report complementary agreement statistics on the 10-case test set. Spearman’s rank correlation was ρ = 0.875, and Lin’s concordance correlation was ρc = 0.839, with an accuracy component Cb = 0.911, indicating the degree of absolute agreement relative to the 45° identity line. A Bland–Altman analysis showed a mean bias of +0.52 rubric points, with 95 percent limits of agreement from −1.61 to +2.66 rubric points. The model prediction vs. the examiner average scores for the testing dataset are shown in Figure 8, while the Bland–Altman plot for the testing dataset is shown in Figure 9.

In terms of accuracy, 50% of predictions fell within ±1 point (equivalent to a ±5% deviation on the 20-point rubric), and 100% fell within ±2 points (±10%), meeting the predefined threshold for educationally acceptable scoring variance. These results confirm the model’s ability to generalize beyond the training data and provide consistent scoring, reinforcing its promise as a supportive tool for preclinical assessment in dental education.

Presented in Table 2 is the detailed scoring per rubric item comparing both the predicted scores (model scores) and the human scores (actual scores) for the 10 model testing cavity preparations.

## 5. Discussion

This study builds on gaps previously identified, offering one of the first documented examples of a dental educator independently developing a neural network model using ChatGPT as a real-time coding tutor. It also represents an initial application of AI for assessing 3D preclinical cavity preparations, demonstrating a novel integration of clinical expertise with LLM-guided development in dental education.

The study provides insights into the feasibility, educational relevance, and future potential of AI-driven assessment tools developed by educators with minimal programming expertise. What initially seemed like a highly technical and inaccessible task was transformed into a structured, step-by-step learning journey. Through conversational guidance, basic computer skills were converted into practical AI capabilities. ChatGPT supported software setup, troubleshooting, and explained Python code in plain language, lowering the technical barrier and showing LLMs’ value for faculty upskilling and AI access in education [16].

The clinician learned how to process 3D scans, convert them into usable point cloud data, integrate detailed marking rubrics, and train a neural network reflective of expert clinical judgment. The LLM not only clarified AI logic and corrected code but also helped structure the model architecture, link examiner scores using JSON labels, and configure training parameters specific to dental education.

Educators with clinical backgrounds can independently develop AI models aligned with their institutional marking standards and learning objectives. By using freely available tools such as Open3D, Jupyter Notebook, and PyTorch, the model was built at a low cost, making the process both feasible and scalable. This model embedded rubric logic directly into its design, enabling automated scoring aligned with examiner standards. Such clinician-led dataset design ensures that educational goals are preserved, something often overlooked in purely technical implementations.

The educational impact is significant. AI-driven 3D assessment tools have the potential to streamline marking, reduce examiner workload, and support more objective, consistent feedback. These systems reduce examiner inconsistencies and human errors, which naturally arise in subjective assessments. The model supports consistent and objective feedback based on clinically accepted rubrics, which is a step in the right direction toward improving the concern of examiner variability in scores, a problem often cited in preclinical dental assessments [17]. Such consistency is essential in ensuring fairness and transparency in skill-based learning environments and aligns well with the move toward competency-based education frameworks.

While the model shows strong promise, its development was based on a limited dataset of 20 marked preparations. These were sufficient to validate the architecture but not to confirm its generalizability. This phase should therefore be considered an exploratory stage, not a final validation. Future work will focus on expanding the dataset to improve the model’s accuracy, robustness, and applicability to a wider range of preclinical assessments. As neural networks typically improve with larger and more diverse datasets, these steps are critical for broader implementation.

These findings support the feasibility of clinician-led AI scoring workflows in preclinical training, particularly for rapid, rubric-linked feedback and examiner calibration. Integration should begin as formative support alongside human grading, with transparent communication to students and governance to monitor bias and drift.

We do not claim generalizable validity at this sample size. Rather, we show a workable pipeline and early alignment, motivating multi-site dataset expansion and external validation.

We deliberately limited analyses to feasibility endpoints on total scores. Criterion-level interpretability (e.g., saliency/occlusion) was out of scope for this pilot and will be addressed when scaling the dataset, alongside criterion-specific calibration, the augmented sampling of proximal regions, and repeated examiner labels to separate model and rater variance. Accordingly, per criterion results here should be viewed as illustrative rather than validated.

With continued refinement, validation, and ethical integration, clinician-led AI systems like the one developed in this study may soon become vital components of competency-based dental education, offering standardization, scalability, and a deeper alignment between assessment tools and learning outcomes.

## 6. Conclusions

This feasibility study is the first to demonstrate that a dental educator without prior coding experience can independently develop a functional neural network model for 3D cavity assessment using ChatGPT. The model showed a close alignment with expert examiner scores, confirming the technical feasibility of building a neural network model using this method. These findings support the potential for scalable, low-cost, clinician-led AI systems to enhance objectivity, consistency, and fairness in preclinical restorative dentistry education.

Limitations: The dataset size (20 train, 10 test) and single tooth type from one institution constrain the generalizability. The results should be interpreted as a feasibility signal for a possible neural network model architecture created by a clinician without prior coding experience.

## Figures and Tables

**Table 1:** Detailed case-by-case comparison of model predicted vs. true human scores for each rubric criterion, providing insight into model behavior and per domain scoring accuracy. Numbers have been approximated to one decimal point.

**Sample Number**	**1**	**2**	**3**	**4**	**5**	**6**	**7**	**8**	**9**	**10**	**11**	**12**	**13**	**14**	**15**	**16**	**17**	**18**	**19**	**20**
True proximal clearance buccal	1	0	0	0	1	0	1	2	0	1	2	1	2	1	0	0	1	1	0	1
**Predicted proximal clearance buccal**	**1**	**0**	**0.2**	**0.4**	**1.3**	**0.5**	**0.7**	**1.4**	**0**	**0.8**	**1**	**1.1**	**1.3**	**0.2**	**0.4**	**0.2**	**0.9**	**1**	**0.1**	**0.7**
True proximal clearance lingual	2	2	2	2	0	2	2	2	1	0	0	0	2	2	2	0	1	0	1	0
**Predicted proximal clearance lingual**	**1.2**	**1.6**	**1.8**	**1.7**	**0.3**	**1.3**	**1.9**	**1.9**	**0.5**	**0.1**	**0.1**	**0**	**1.1**	**1.9**	**1.9**	**0.3**	**0.7**	**0**	**1**	**0**
True proximal clearance gingival	2	1	2	2	2	2	2	2	0	2	0	2	2	0	2	0	1	1	0	0
**Predicted proximal clearance gingival**	**1.5**	**0.3**	**1.8**	**1.2**	**1.9**	**1.1**	**1.9**	**1.9**	**0.1**	**1.5**	**0.8**	**1.9**	**1.7**	**0.7**	**1.9**	**0.5**	**1.6**	**0.7**	**0.1**	**0.2**
True preservation between occlusal and proximal	2	2	2	2	1	2	2	2	2	2	2	2	2	2	2	2	2	2	2	1
**Predicted preservation between occlusal and proximal**	**1.8**	**1.8**	**1.9**	**1.9**	**1.4**	**1.8**	**1.9**	**1.9**	**1.9**	**1.9**	**1.7**	**1.7**	**1.8**	**1.9**	**1.9**	**1.8**	**1.8**	**1.9**	**1.9**	**1.7**
True occlusal preservation	1	1	1	1	1	1	1	1	1	1	1	1	1	1	1	1	1	1	1	1
**Predicted occlusal preservation**	**0.9**	**0.9**	**0.9**	**0.9**	**0.9**	**0.9**	**0.9**	**0.9**	**0.9**	**0.9**	**0.9**	**0.9**	**0.9**	**0.9**	**0.9**	**0.9**	**0.9**	**0.9**	**0.9**	**0.9**
True caries removal	8	9	9	9	9	7	9	9	9	9	9	9	7	9	9	9	9	9	7	7
**Predicted caries removal**	**8.1**	**8.6**	**8.9**	**8.7**	**8.5**	**8.3**	**8.9**	**8.9**	**8.9**	**8.9**	**8**	**8.8**	**7.3**	**8.7**	**8.9**	**8.7**	**8.5**	**8.7**	**7.1**	**7.2**
True no undermined enamel	2	1	2	2	2	2	2	2	0	1	0	1	1	2	2	0	2	0	0	0
**Predicted no undermined enamel**	**1.7**	**0.9**	**1.8**	**1.8**	**1.6**	**1.4**	**1.9**	**1.9**	**0**	**0.4**	**0.3**	**0.6**	**0.9**	**1.8**	**1.9**	**0.1**	**1.6**	**0**	**0**	**0**
True damage to adjacent teeth	0	0	0	0	0	0	0	0	0	0	0	0	0	0	0	0	0	0	0	0
**Predicted damage to adjacent teeth**	**0**	0	0	0	0	0	0	0	0	0	0	0	0	0	0	0	0	0	0	0
True total	18	16	18	18	16	16	19	20	13	16	14	16	17	17	18	12	17	14	11	10
**Predicted total**	**16.6**	**14.6**	**17.7**	**17**	**16.3**	**15.6**	**18.7**	**19.4**	**12.7**	**14.8**	**13.2**	**15**	**15.3**	**16.3**	**18.4**	**12.9**	**16.5**	**13.5**	**11.4**	**11.19**

**Table 2:** Detailed case-by-case comparison of model predicted vs. human true scores for each rubric criterion, providing insight into model behavior and per domain scoring accuracy.

**Sample Number**	**1**	**2**	**3**	**4**	**5**	**6**	**7**	**8**	**9**	**10**
True proximal clearance buccal	0.5	0	0	0	0	0	0	0	0	0
**Predicted proximal clearance buccal**	**0**	**0**	**0**	**0**	**0**	**0**	**0**	**0.3**	**0**	**0**
True proximal clearance lingual	0	0	0	1	2	0	0	1	0	0
**Predicted proximal clearance lingual**	**0**	**0**	**0**	**0**	**0**	**0**	**2**	**0.4**	**0**	**2**
True proximal clearance gingival	0	0	0	0	0	0	0	0	1	1
**Predicted proximal clearance gingival**	**0**	**0.6**	**0**	**0**	**0**	**1**	**0**	**0.3**	**0**	**0**
True preservation between occlusal and proximal	2	2	1	2	0	1	2	1	2	2
**Predicted preservation between occlusal and proximal**	**2**	**2**	**2**	**2**	**2**	**2**	**2**	**2**	**2**	**2**
True occlusal preservation	1	1	1	1	0.5	0	1	0	1	2
**Predicted occlusal preservation**	**1**	**1**	**1**	**1**	**1**	**1**	**1**	**1**	**1**	**1**
True caries removal	7	8	7	7	8	8	9	6	4	8
**Predicted caries removal**	**8.5**	**9**	**4.7**	**6**	**7.4**	**3.2**	**8.8**	**2.2**	**5.4**	**8.1**
True no undermined enamel	1	0	0	0	0	0	2	0	1	0
**Predicted no undermined enamel**	**0**	**0**	**0**	**0**	**0**	**1.28**	**1.9**	**0**	**0**	**0**
True damage to adjacent teeth	**0**	0	0	−2	−1	−1	0	0	0	−1
**Predicted damage to adjacent teeth**	**0**	0	0	0	0	0	0	0	0	0
True total	11.5	11	9	9	9.5	8	12	8	9	12
**Predicted total**	**11.5**	**12.6**	**7.7**	**9**	**10.4**	**8.5**	**13.8**	**6.3**	**8.4**	**13.1**

## Data Availability

The datasets and AI model developed for this study are not publicly available. Researchers interested in academic collaboration or non-commercial use may contact the corresponding author. Requests will be reviewed on a case-by-case basis and may require a data use agreement. A sample dataset and a sample of the chat with ChatGPT may be accessed through the following link: https://osf.io/3k5uv/overview accessed on 29 October 2025.

## Code Availability

The code was developed by the primary author with real-time support from ChatGPT. Academic access may be considered upon request to the corresponding author. A sample of a Jupyter notebook code cell is available in the manuscript. Requests will be reviewed on a case-by-case basis and may require a data use agreement.
